# Epidemiology of drowning in children and adolescents survived by Guilan's EMS from 2016 to 2017

**Published:** 2019-07

**Authors:** Payman Asadi, Fatemeh Niazmand, Seyedeh-Masoomeh Maleki-Ziabari

**Affiliations:** ^*a*^Disaster & Emergency Management Center, Guilan University of Medical Sciences, Rasht, Iran.

## Abstract

**Background:**

Falling into water and drowning is one of the preventable events resulting in neurological damages and death due to hypoxia and ischemia in children and adolescents. This epidemiological study aimed at investigating drowning cases among children and adolescents rescued by the Guilan's EMS from 2016 to 2017.

**Methods:**

This is a cross-sectional study in which 160 cases of drowning were rescued by EMS. A checklist including age, sex, the location of drowning (river, swimming pool, out of designed zone at beach, among others), the status of patient (inpatient, outpatient, death) was filled out. The data were analyzed by SPSS 19 , chi square and t-test.

**Results:**

Of all 160 cases of drowning in Guilan from 2016 to 2017, 74.3% were men and 25.6% women. The minimum and maximum age of drowned cases were 2 and 82 years old, respectively (mean age =27 years old). Most of the drowning cases (87.5%) occurred out of the patrolled areas of the beach. 8.7% of the victims were drowned in the rivers. Of all the recorded drowned cases, 16.8% died, 61.8% were hospitalized and 21.3% were outpatient.

**Conclusions:**

As the number of drowning cases has increased 7.7% in 2017 compared to 2016 and children under 5 years of age (1-5 years old) are among high risk groups of drowning, they should be focused for prevention strategies. Authorities in charge should prioritize the issues of controlling and prevention of such events for children.

**Keywords:**

Drowning, Children, Adolescents, EMS

